# Optimized Dosing Regimens of Meropenem in Septic Children Receiving Extracorporeal Life Support

**DOI:** 10.3389/fphar.2021.699191

**Published:** 2021-08-24

**Authors:** Yixue Wang, Weiming Chen, Yidie Huang, Guangfei Wang, Zhiping Li, Gangfeng Yan, Chao Chen, Guoping Lu

**Affiliations:** ^1^ Department of Pediatric Critical Care Medicine, Children’s Hospital of Fudan University, National Children’s Medical Center, Shanghai, China; ^2^ Department of Clinical Pharmacy, Children’s Hospital of Fudan University, National Children’s Medical Center, Shanghai, China; ^3^ Department of Neonatology, Children’s Hospital of Fudan University, National Children’s Medical Center, Shanghai, China

**Keywords:** meropenem, pharmacokinetics/pharmacodynamics, children, sepsis, even extracorporeal membrane oxygenation, continuous renal replacement therapy, target attainment, estimated creatinine clearance

## Abstract

**Objectives:** To develop a population pharmacokinetic model of meropenem in children with sepsis receiving extracorporeal life support (ECLS) and optimize the dosage regimen based on investigating the probability of target attainment (PTA).

**Methods:** The children with sepsis were prospectively enrolled in a pediatric intensive care unit from January 2018 to December 2019. The concentration-time data were fitted using nonlinear mixed effect model approach by NONMEM program. The stochastic simulation considering various scenarios based on proposed population pharmacokinetics model were conducted, and the PTAs were calculated to optimize the dosage regimens.

**Results:** A total of 25 children with sepsis were enrolled, of whom13 received ECMO, 9 received CRRT, and 4 received ECMO combined with CRRT. 12 children received a two-step 3-h infusion and 13 children received 1-h infusion. Bodyweight and creatinine clearance had significant impacts on the PK parameters. ECMO intervention was not related to the PK properties. If 100%T > MIC was chosen as target, children receiving 40 mg/kg q8h over a 3 h-infusion only reached the PTA up to 77.4%. If bacteria with MIC 2 mg/L were to be treated with meropenem and the PTA target was 50%T > MIC, a dose of 40 mg/kg q8h for 1 h infusion would be necessary.

**Conclusions:** The PK properties of meropenem in septic children receiving extracorporeal life support were best described. We recommended the opitimized dosing regimens for septic children receiving ECLS depending on the PTA of PK target 50%T > MIC and 100%T > MIC, for children with sepsis during ECLS with different body weight, estimated creatinine clearance (eCRCL) and MIC of bacteria.

## Introduction

For Severe infection is a leading cause of hospital admission and a common cause of mortality in the Intensive Care Unit (ICU) and Pediatric Intensive Care Unit (PICU) (Kempker and Martin 2016). β-lactam antibiotics are the most commonly prescribed drugs for septic children with numerous clinical indications. Yet, dosing and infusing time of certain β-lactam antibiotics, such as meropenem, are still controversial in septic patients. Although septic children receive broad-spectrum antibiotics, blood purification therapy and even extracorporeal membrane oxygenation (ECMO) (Rhodes et al., 2017), their mortality rate can reach approximately 25% worldwide (Menon and Wong 2015; Mathias et al., 2016), while the antimicrobial effect is not often ideal and individual differences exist.

The probability of target attainment (PTA) of the dosing interval during which the free drug concentration exceeds the minimal inhibitory concentration (%T > MIC) has been proven to be correlated with clinical improvement in patients treated with meropenem (Ariano et al., 2005; Li et al., 2007; Crandon et al., 2016). A recent report showed the high inter- and intra-patient variability in meropenem concentrations in adults suffering from sepsis and acute kidney injury (AKI), with the attainment of the target 100%T > MIC and 50%T > 4 × MIC of 48.4 and 20.6%, respectively (Ehmann et al., 2017). Meropenem is a small hydrophilic molecule with a low volume of distribution (Vd) (0.3 L/Kg), very low protein binding (<2%), high affinity for water, low Vd and high unbound fraction, which can be easily eliminated by the kidney (Agencia Española de Medicamentos y Productos Sanitarios; Meronem IV 500 mg & 1 g, 2017; Mattoes et al., 2004; Sanitarios, Turnidge 1998; Ulldemolins et al., 2014). The high inter- and intra-patient variability in meropenem concentrations suggested that individual therapy was needed for meropenem treatment in order to achieve target concentration.

There has been limited data on whether ECLS influences PK/PD of meropenem in critically ill children (Cies et al., 2014; Nehus et al., 2016). There are various settings and membrane characteristics in continuous renal replacement therapy (CRRT). At present, it remains unclear whether the type of hemofiltration membrane would affect the clearance rate of β-lactam antibiotics. ECMO is a supportive therapy in septic shock and severe circulation failure in children and adults. A meta-analysis in adults demonstrated that ECMO did not influence PK of non-lipophilic drugs (Abdul-Aziz et al., 2017), which was in accord with our reported PK parameters of meropenem in children receiving 20 mg/kg q8h meropenem (Wang et al., 2021). But Shekar et al. found that variability in meropenem clearance was correlated with creatinine clearance or the presence of CRRT (Shekar et al., 2014). Wang et al. demonstrated that standard dosage regimens for meropenem did not meet an appropriate PD target but they did not do research on children undergoing extracorporeal life support (ECLS) (Wang et al., 2020).

The aim of our study was to develop a population PK model of meropenem in children with sepsis receiving extracorporeal life support and assess their probability of target attainment by MIC, in order to provide optimal dosing recommendations based on clinical characteristics. This is a continued PK modeling study of our published one (Wang, Li, Chen, Yan, Wang, Lu and Chen 2021).

## Methods

### Ethics

All the children enrolled in the study have been provided consent for care. The Ethics Committees of Children’s Hospital of Fudan University approved the current study (2016-133, 2016-311). For study participation, both parents signed a written consent. A data safety and monitoring board reviewed results after half of participants were enrolled.

### Clinical Study

This was a prospective observational study conducted at the PICU of Children’s Hospital of Fudan University, Shanghai, China, between January 2018 and December 2019. The protocol was registered on the Chinese Clinical Trial Registry (http://www.chictr.org.cn/index.aspx, ChiCTR-OPC-16008703).

The inclusion and exclusion criteria were shown in Table 1. MICs were determined in the microbiology laboratory of Children’s Hospital of Fudan University (Shanghai, China). MICs for meropenem were evaluated by a diffusion method on a solid-state culture, using the E-test methodology. When the MIC was not available, epidemiological cut-off (ECOFF) was decided by the European Committee on Antimicrobial Susceptibility Testing (EUCAST) data (Polsfuss et al., 2012).

**TABLE 1:** Inclusion and exclusion criteria.

Inclusion criteria
● Age from 29 days to 18 years diagnosed with sepsis (defined according to surviving sepsis campaign guidelines^18^)
● Clinical indication for intravenous meropenem
● Presence of intra-arterial line for blood sampling or CRRT filter port sampling is possible
● Informed consent available from both patients
**Exclusion criteria**
● Critically ill patients with poor prognosis (not expected to survive through sampling schedules)
● Unable to obtain informed consent
● Failure to open intra-arterial line access and CRRT filter port sampling isn’t possible

Meropenem (Meropen® injection; Sumitomo Pharmaceuticals (Suzhou) Co., Ltd., produced in Osaka, Japan) was administered q8h intravenously for 1 h or 3 h (two step infusion). Dosing 20 mg/kg or 40 mg/kg q8h decided by the responsible clinical team independently was administered intravenously for 60 min or infused by two steps. The two-step infusion is the prolonged infusion for which half the dose is infused in the first 0.5 h, and the other half is continuously infused in the next 2.5 h. The intensive PK samples were collected after at least 5 doses of meropenem. For children with BW ≤ 30 kg, the blood samples were then collected from a peripheral arterial catheter or CRRT filter port at 0 (pre-dose), 5, 45, 90, 180, 360, and 480 min after the end of infusion of meropenem. For children with BW > 30 kg, the blood samples were collected at 0 (pre-dose), 5, 15, 30, 60, 90, 120, 240, 360, and 480 min after the end of infusion of meropenem. For each children receiving CRRT, the total volume of effluent fluid of CRRT was recorded, and 10 ml of effluent liquid was stored at −80°C. All the blood and effluent fluid samples were stored at −80° for ≤7 days before the concentration analysis. The method for measuring the concentration of effluent liquid was the same as the blood samples.

### Data Collection

Plasma and effluent fluid concentrations of meropenem were analyzed with High-Performance Liquid Chromatography tandem mass spectrometry (HPLC-MS/MS) at the center laboratory of the School of Pharmacy, Fudan University (Shanghai, China). (See Supplementary Datasheet S1: Bioanalytical method for meropenem concentration). The following data was recorded in a pre-defined CRF for each enrolled child: age, gender, weight, height, Weight Adjusted Z scores (WAZ) (Black et al., 2013), Severity of disease (Pediatric Risk of Mortality score Ⅲ (PRISM-Ⅲ) on the day of admitting to PICU), creatinine, nitrogen urea, bilirubin, albumin, glutamic-pyruvic transaminase/glutamic oxaloacetic transaminase (GPT/GOT), eCRCL derived from the Schwartz formula, positive bacteria culture results, ECMO and CRRT settings (See Supplementary Datasheet S2: Original data for model analysis). The BioPump CBBPX-80 (Medtronic Biomedicus, Inc., Minneapolis, MN, United States) was used in all children receiving ECMO. The oxygenator and tubing used was the Oxygenator D905 EOS or LILLIPUT 2 ECMO manufactured by Sorin Group Italia, Mirandola, Italy (LivaNova, London, United Kingdom). CRRT was performed with continuous venovenous hemodialysis (CVVHDF) mode using the filter with AN69 membrane (Baxter, United States) through a Prismaflex CRRT system (Baxter, United States). Overall, 10 microorganisms were documented in clinical samples. The main documented microorganisms were: *Pseudomonas aeruginosa* (*n* = 4, 40%) (meropenem MIC≤1 ug/ml in 4 cases), Burkholderia cepacia (*n* = 3, 30%) (meropenem MIC≤1 ug/ml in 3 cases), *Klebsiella pneumoniae* (*n* = 1, 10%) (MIC = 2 mg/ml in 1 case), Klebsiella aerogenes (*n* = 1, 10%) (meropenem MIC≤1 ug/ml in 1 cases) and Flavobacterium meningesepsis (*n* = 1, 10%) (meropenem MIC≤1 ug/ml in 1 cases).

### Population Pharmacokinetics

Twenty-five children were enrolled in the population PK modeling analysis. A total of 225 blood samples were available for meropenem concentration measurements, and 35 out of 225 (15.6%) meropenem plasma samples were below the LLOQ of 0.2 μg/ml.

The population PK analysis was performed using nonlinear mixed-effects modeling in the NONMEM® program (version 7.4, ICON Development Solutions, Ellicott City, MD, United States); gFortran (version 4.60). Perl-speaks-NONMEM (PsN; version 4.6.0) and R language (version 3.2.0, http://www.r-project.org/) were used to evaluate the goodness of fit and output visualizations. Part of the parameters of non-compartmental analysis was published as mentioned previously (Wang et al., 2021), and this article only reported the result of model analysis. The first-order conditional estimation algorithm with η-ε interaction (FOCE-I) was used throughout the model-building procedure. Discrimination between models during the model building process was based on the objective function value (OFV), and calculated as proportional to twice the log-likelihood (−2LL). A reduction in OFV (∆OFV) of 3.84 was considered a significant improvement (*p* < 0.05) between two hierarchical models after the inclusion of one additional parameter or inter-individual variability (one degree of freedom difference).

Different methods were used to investigate the influence of data censoring below the LLOQ. Data measuring below the LLOQ (refer to M1-method) were excluded, thus maximizing the likelihood to predict censored data to be below LLOQ (refer to M3-method), and imputing the first concentration below LLOQ within a patient as half of the LLOQ (refer to M6-method) were evaluated. The predictive performance of the different methods was assessed by comparing the proportion of predicted and observed data censoring below the LLOQ, using categorical visual predictive checks (Ahn et al., 2008).

The meropenem plasma concentrations were transformed with natural logarithm. First-order elimination of meropenem was assumed to occur from the central compartment.

The model development process was performed using three steps. First, a base model without incorporating covariates was selected. In this step, all possible structural compartments were investigated, i.e., one, two, and three-compartment disposition models.

**FIGURE 1:**
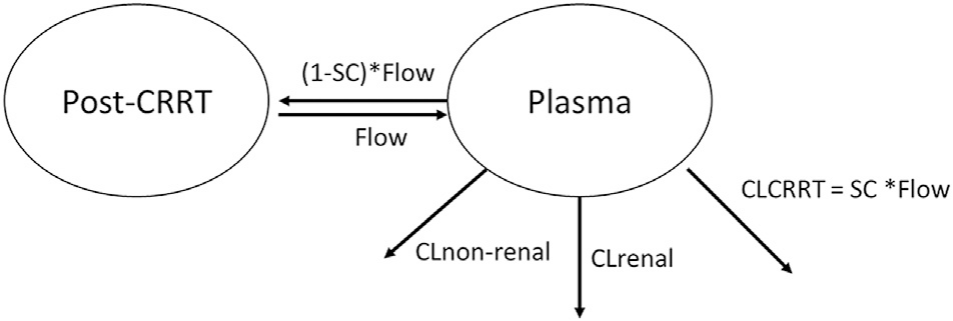
Graphical overview of the structural pharmacokinetic model for meropenem. CL stands for clearance. CLCRRT stands for the clearance regarding CRRT. SC stands for the sieving coefficient. Flow is the sum of replacement flow and filtration flow.

The clearance in children without CRRT intervention was described in Equation (1). For the pediatric patients receiving CRRT intervention, the concentrations of meropenem post dialysate-ultrafiltrate were modeled by adding a post-CRRT compartment with a parameter, sieving coefficient (SC), which was defined as the fraction the drug eliminated across the membrane during CRRT, as shown in Figure 1. The clearance (CL) parameter was described in Equation (2) by adding a CRRT clearance further.

$$\text{CL}\;=\;\theta_{1}$$ (1)

$$\text{CL}\;=\;\theta_{1}+\text{SC}\cdot \text{Flow}$$ (2)

where the flow (intercompartment clearance, Q) was the sum of the replacement and filter flows, 45 ml/kg/h in this study. The average volume of the post-CRRT compartment (V2) was 25 ml, which is the volume of pipeline where the efflux sample was collected. The Q and V2 were derived from CRRT configuration, and therefore, fixed in the modeling analysis.

Inter-individual variability was exponentially added to all parameters (Equation (3)).

$$\theta_{i}=\;\theta \cdot \exp\left(\eta_{i,\theta }\right)$$ (3)

where, $\theta_{i}$ is the individual parameter estimate for the ith individual, θ is the population estimate of the investigated parameter, and $\eta_{i,\theta }$ is the inter-individual variability (IIV) of the investigated parameter, assumed to be normally distributed with a zero mean and variance ω2.

The unexplained residual variability, that was assumed to be normally distributed with a zero mean and variance σ2, was modeled with an additive error on the natural log-transformed concentrations, which is approximately equivalent to an exponential residual error on an arithmetic scale. The separate residue errors were used for plasma and post dialysate-ultrafiltrate concentration, respectively.

### Covariate Modeling

First, the non-CRRT CL parameter ($\theta_{1}\;$in Equations (1) and (2)) was described further as the sum of the non-renal CL (not affected by any of the explored covariates), and renal CL (affected by CLCR, the second part in Equation (2)), as shown in Equations (4) and (5).

$$\text{CL}\;=\;\theta_{1}\cdot \left(1+\left(\text{CRCL}-150\right)\cdot \theta_{2}\right)$$ (4)

Then Equation (4) could be further written to:

$$\text{CL}\;=\;\theta_{1}+\theta_{1}\cdot \left(\text{CRCL}-150\right)\cdot \theta 2$$ (5)

Second, the body weight was added in the model as simultaneous incorporation of an allometric function on all clearance and distribution volume parameters (Wang et al., 2012; Holford et al., 2013), but except for V2 and Q, respectively (Equations (6) and (7)).

$${\text{CL}}_{i}=\;{\text{CL}}_{\text{typical}}\cdot {\left(\frac{{\text{BW}}_{i}}{{\text{BW}}_{\text{median}}}\right)}^{0.75}\cdot \exp\left(\eta_{i,\text{CL}}\right)$$ (6)

$$V_{i}=\;V_{\text{typical}}\cdot \left(\frac{{\text{BW}}_{i}}{{\text{BW}}_{\text{median}}}\right)\cdot \exp\left(\eta_{i,V}\right)$$ (7)

where BWi is the individual bodyweight, and BW median is the median bodyweight of the PK population (i.e., 12 kg) in this study.

The age-related maturation effect on CL was investigated further Equation (8).

$${\text{CL}}_{i}={\text{ CL}}_{\text{typical}}\cdot \left(\frac{{\text{Age}}_{i}}{{\text{Age}}_{i}+{\text{Age}}_{50}}\right)\cdot {\left(\frac{{\text{BW}}_{i}}{{\text{BW}}_{\text{median}}}\right)}^{0.75}$$ (8)

where, Age_i_ is the individual age, age_50_ was the age to reach 50% full CL maturation.

Finally, other covariates (e.g., receiving ECMO, nutrition status, liver function, gender, concomitant drugs) were investigated for all model parameters. The analysis was performed with a stepwise forward additive approach followed by a stepwise backward elimination approach with *p*-values of <0.05 and 0.001, respectively. Uncorrelated covariates were included in the model using different functional forms like linear, power and exponential functions.

### Model Evaluation

Basic goodness-of-fit diagnostics were used to evaluate systematic errors and model misspecification. The sampling importance resampling (SIR) approach was used to calculate parameter uncertainty in the final population PK model (samples = 2,000, resamples = 1,000). The overall predictive performance of the final model was evaluated using simulation-based diagnostics (i.e., visual predictive checks, *n* = 2,000 simulations).

### In Silico Simulation

The silico simulations were performed to generate the concentration-time course of meropenem, based on the final population PK model. The various simulation scenarios were based on bodyweight (5, 10, 20, 30 kg), infusion time (1 and 3 h), eCRCL (30, 60, and 90 ml/min). For the patients with normal renal function (i.e., eCRCL was 90 ml/min), only the renal and non-renal intrinsic clearance were considered in the simulation. However, for the patients with impaired renal function (i.e., eCRCL were 30 or 60 ml/min), apart from the intrinsic clearance, the CRRT clearance was also considered in the simulation (refer to Equation (2) above).

Sequentially, the fraction of time during the dosing interval that drug concentrations remain above MIC (f%T > MIC) was calculated. The probability of target attainment (PTA) for achieving 50 and 100% f%T > MIC were also assessed.

## Results

The demography of included pediatric patients is shown in Table 2. Our study included 25 children diagnosed with sepsis, among whom, 13 patients received ECMO, 9 received CRRT, 4 received ECMO combined with CRRT, and 7 patients received neither ECMO nor CRRT. Three children received meropenem at a dose of 40 mg/kg q8h, while the others received meropenem at a dose of 20 mg/kg q8h. Twelve patients received an two-step 3-h intravenously infusion and 13 patients received 1-h infusion. Apart from renal function parameters (SCR and CLCR), the demographic parameters of pediatric patients with and without ECLS (i.e., ECMO, CRRT, or their combination) did not significantly differ.

**TABLE 2:** The demography of septic pediatric patients.

Characteristics	ECLS *n* = 13	Non-ECLS *n* = 12	All *n* = 25
Age (years)	2.25 (0.69, 4.25)	1.25 (0.67, 2.50)	2.00 (0.71, 3.88)
Bodyweight (kg)	15.26 (8.83)	11.00 (7.00, 13.00)	11.50 (9.50, 36.30)
Height (cm)	93.86 (26.67)	78.00 (70.00, 87.00)	82.00 (71.50, 112.50)
WAZ	0.21 (1.43)	-0.27 (2.02)	0.04 (1.61)
BUN (mmol/L)	9.65 (7.52)	4.23 (2.23)	5.10 (3.15, 8.50)
SCR (µmol/L)	46.00 (25.00, 107.80)	20.00 (14.00, 26.00)	26.00 (19.50, 75.00)
ALT (U/L)	30.75 (9.85, 82.00)	39.00 (16.00, 134.00)	36.60 (12.40, 84.00)
AST (U/L)	82.75 (36.50, 268.50)	54.40 (17.50, 102.00)	65.00 (31.70, 174.70)
DBIL (µmol/L)	4.50 (2.88, 16.63)	3.40 (2.10, 12.20)	4.40 (2.60, 15.75)
TBIL (µmol/L)	14.90 (6.43, 36.75)	8.00 (4.80, 17.00)	13.50 (6.15, 35.35)
Albumin (g/L)	36.60 (6.63)	34.64 (6.41)	35.16 (6.80)
Globulin (g/L)	22.43 (6.47)	25.53 (4.94)	23.46 (6.34)
Female, n (%)	7 (53.84)	5 (41.67)	12 (48.00)

Notes: The eCRCL of CRRT groups was significantly lower than that of the non-CRRT groups. At the same time, the SCR of CRRT groups was significantly higher than that of the non-CRRT groups. The other aspects of characteristics of the four groups showed no significant difference. WAZ: weight for age Z score. TBIL: total bilirubin; DBIL: direct bilirubin; eCRCL: creatinine clearance; SCR: serum creatinine.

The base model using one compartment with first-order elimination had an OFV of 187.842, with the clearance and V estimates of 9.59 L/h (IIV = 0.47) and 23.2 L (IIV = 0.504), respectively. Incorporation of the post-CRRT compartment improved the model fit significantly (∆OFV = −4.924), which was further improved by adding eCRCL on the non-CRRT clearance (∆OFV = −4.419). The structure of the final model is shown in Figure 1.

Bodyweight, implemented as a fixed allometric function on all clearance and volume of distribution parameters, showed a substantial improvement in model fit (∆OFV = −12.039). Age did not have a further significant impact on the pharmacokinetic properties of meropenem. Inclusion of other covariates (e.g., ECMO intervention, WAZ, liver function, concomitant drug) did not significantly affect the PK properties. WAZ is the number of standard deviations of the actual weight of a child from the median weight of the children of his/her age as determined from the standard sample. It did not influence the PK properties of meropenem albeit it reveals the nutrition status of children. The covariates screening process is described in Table 3.

**TABLE 3:** The model building and covariates screening process.

Model No	Model building procedure	Relation	Ofv	Delta OFV	Significance
	Model structure				
1	Base model		187.842		
2	Base model + CRRT		182.918	−4.924	Yes
Separate non-renal and renal clearance (based on model 2)					
3	eCRCL-CL	Linear	178.769	−4.419	Yes
Allometric scaling of bodyweight (based on model 3)					
4	WT-CL and V1	Power	166.73	−12.039	Yes
Other covariates screening (based on model 4): Forward inclusion followed by backward elimination					
Forward inclusion					
5	CL-ALB	Linear	166.64	−0.094	No
6	CL-ALT	Linear	166.63	−0.099	No
7	CL-AST	Linear	166.73	0.000	No
8	CL-DBIL	Linear	166.61	−0.122	No
9	CL-ECMO	Linear	166.52	−0.214	No
10	CL-GLB	Linear	166.42	−0.305	No
11	CL-HT	Linear	166.70	−0.033	No
12	CL-LIZ	Linear	161.26	−5.466	Yes
13	CL-SEX	Linear	165.58	−1.154	No
14	CL-TBIL	Linear	166.66	−0.072	No
15	CL-VANC	Linear	164.31	−2.419	No
16	CL-WAZ	Linear	166.04	−0.695	No
17	SC-ALB	Linear	165.56	−1.174	No
18	SC-ALT	Linear	166.48	−0.249	No
19	SC-AST	Linear	166.73	0.000	No
20	SC-DBIL	Linear	166.38	−0.354	No
21	SC-ECMO	Linear	166.23	−0.504	No
22	SC-GLB	Linear	166.50	−0.226	No
23	SC-HT	Linear	166.64	−0.090	No
24	SC-LIZ	Linear	165.91	−0.825	No
25	SC-SEX	Linear	166.73	−0.004	No
26	SC-TBIL	Linear	166.35	−0.385	No
27	SC-VANC	Linear	166.73	−0.005	No
28	SC-WAZ	Linear	165.41	−1.321	No
29	V1-ALB	Linear	166.73	0.000	No
30	V1-ALT	Linear	166.73	0.000	No
31	V1-AST	Linear	166.73	0.000	No
32	V1-DBIL	Linear	166.68	−0.046	No
33	V1-ECMO	Linear	166.66	−0.068	No
34	V1-GLB	Linear	166.58	−0.149	No
35	V1-HT	Linear	166.58	−0.151	No
36	V1-LIZ	Linear	166.62	−0.111	No
37	V1-SEX	Linear	165.87	−0.856	No
38	V1-TBIL	Linear	166.22	−0.505	No
39	V1-VANC	Linear	166.68	−0.052	No
40	V1-WAZ	Linear	166.45	−0.278	No
Backward elimination					
41	CL–LIZ	Linear	166.73	5.466	No
Residual error model (based on model 4)					
42	2 residue error for plasma and post-dialysis concentration		159.336	−7.394	Yes

Notes: WAZ: weight-age Z score,ALB: albumin, GLB: globulin, TBIL: total bilirubin; DBIL: direct bilirubin; eCRCL: estimated creatinine clearance, VANC: vancomycin, LIZ: linezolid.

**FIGURE 2:**
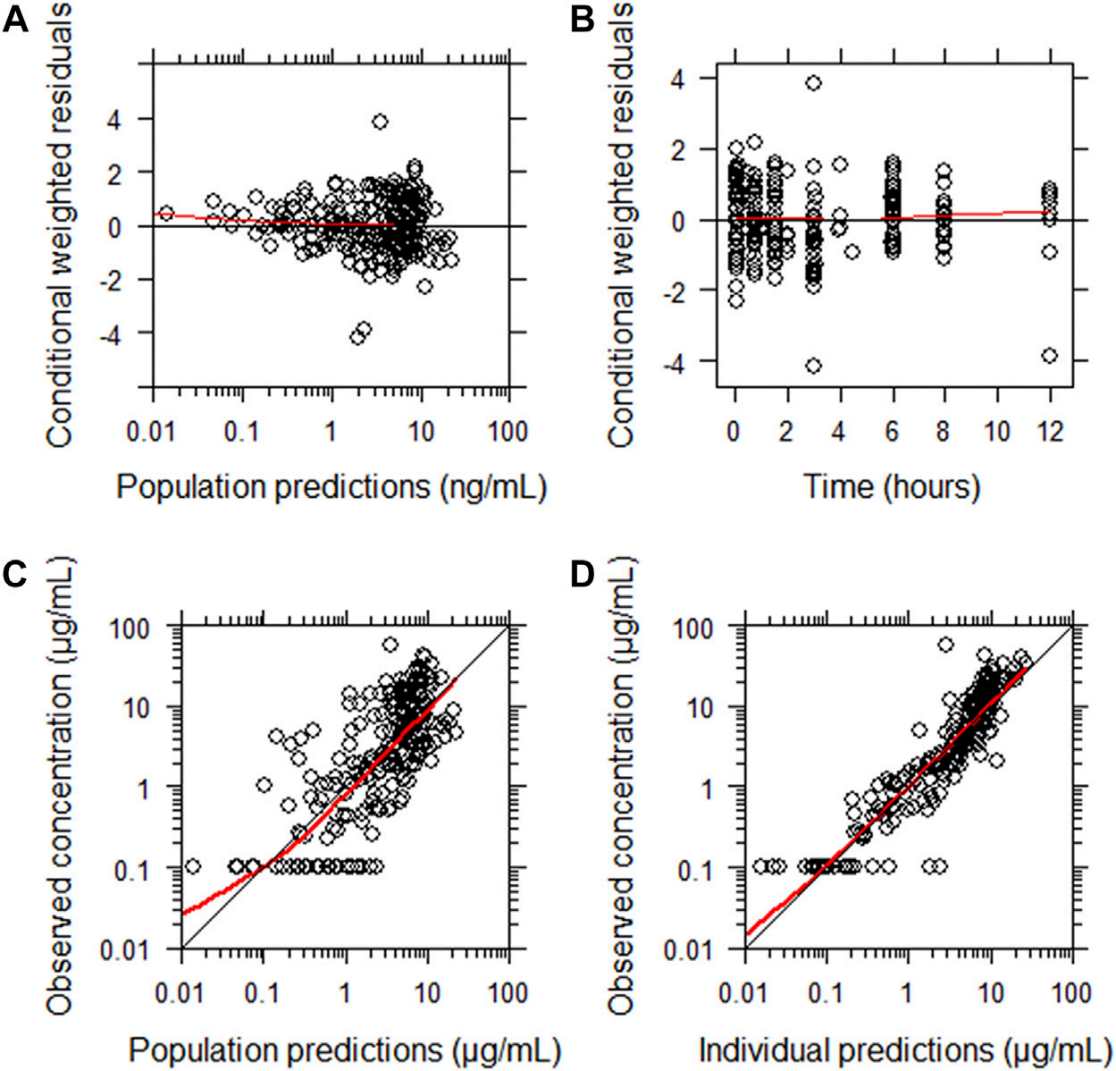
Goodness-of-fit of the final population pharmacokinetic model describing meropenem. **(A)** Conditionally weighted residuals vs population predicted concentrations. **(B)** Conditionally weighted residuals vs time. **(C)** Observed plasma concentrations vs population predicted concentrations. **(D)** Observed plasma concentrations vs individually predicted concentrations. Solid red lines represent locally weighted least squares regressions. There is consistency around lines of unity and a lack of bias noted.

The final parameter estimates showed good precision with relatively small standard errors (Table 4), confirming the stability of the model and providing confidence when using the developed population PK model to simulate different adherence scenarios. Goodness-of-fit diagnostic plots (Figure 2) demonstrated good description of observed data.

**TABLE 4:** Final population PK parameter estimates of meropenem in septic children.

Parameters	NM estimates	SIR median (95%CI)	CV for IIV	SIR median (95%CI)
CL (L/h)	7.6 (13.3)	7.6 (5.9–9.7)	55.7 (21.8)	56.4 (41.5–77.1)
V1 (L)	21.4 (16.5)	21.3 (16.5–29.0)	56.2 (26.9)	57.2 (36.2–83.0)
Q (L/h/kg)	0.045 * bodyweight fixed	—	—	—
SC (%)	25.7 (28.4)	25.7 (10.4–40.5)	—	—
V2 (L)	0.025 fixed	—	—	—
CRCL on CL (min·1.73 m^2^/ml)	0.0035 (19.8)	0.0035 (0.17–0.61)	—	—
RUV for plasma	0.575 (36.0)	0.571 (0.448–0.767)	—	—
RUV for efflux CRRT	0.284 (17.9)	0.287 (0.204–0.410)	—	—

Notes: CL, Clearance; V1 is central volume; Q is the inter-compartmental clearance between central compartment and post-CRRT compartment, V_2_ is the volume of post-CRRT compartment, RUV is the residual error on log scale.

Clearance was described by followed equation: for children not receiving CRRT then $\mathrm{CL=7}\mathrm{.6}\cdot {\left({\text{BW}}_{i}/12\right)}^{0.75}$ (1+(eCRCL-150) 0.0035), for the children undergoing CRRT, then$\mathrm{CL=7}\mathrm{.6}\cdot {\left({\text{BW}}_{i}/12\right)}^{0.75}$ ∙ (1+(eCRCL-150)∙0.0035)+0.257∙Q). $\mathrm{VI=21}\mathrm{.4}\left({\text{BW}}_{i}/{\text{BW}}_{12}\right)$. Where, BWi is the individual bodyweight.SIR: The sampling importance resampling method. The uncertainty was derived from SIR with options of 2000 samples and 1,000 resamples.

**FIGURE 3:**
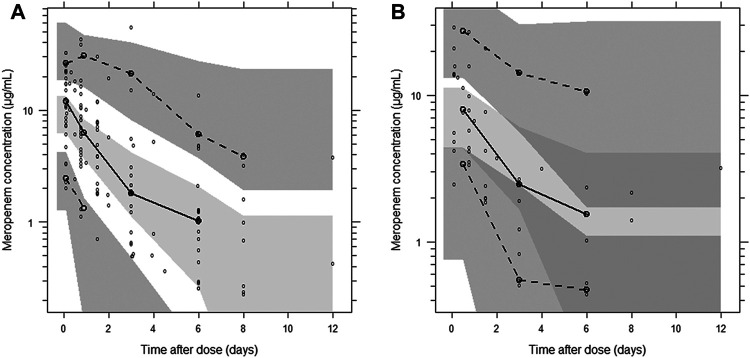
Visual predictive check of the final population pharmacokinetic model for meropenem. **(A)** Plasma concentration; **(B)** post-CRRT concentration. Based on 1,000 stochastic simulations. Open circles represent the observations, and solid lines represent the 5th, 50th, and 95th percentiles of the observed data. The shaded areas represent the 95% confidence intervals around the simulated 5th, 50th, and 95th percentiles.

A categorical visual predictive check for censored data showed good agreement between predicted and observed censoring concentration data below LLOQ when using the forementioned M6 approach. The use of a more sophisticated approach to handle LLOQ data, like M3 method (likelihood estimation), was not necessary and therefore not considered further. The visual predictive checks demonstrated the adequate predictive performance of the final model (Figure 3).

**FIGURE 4:**
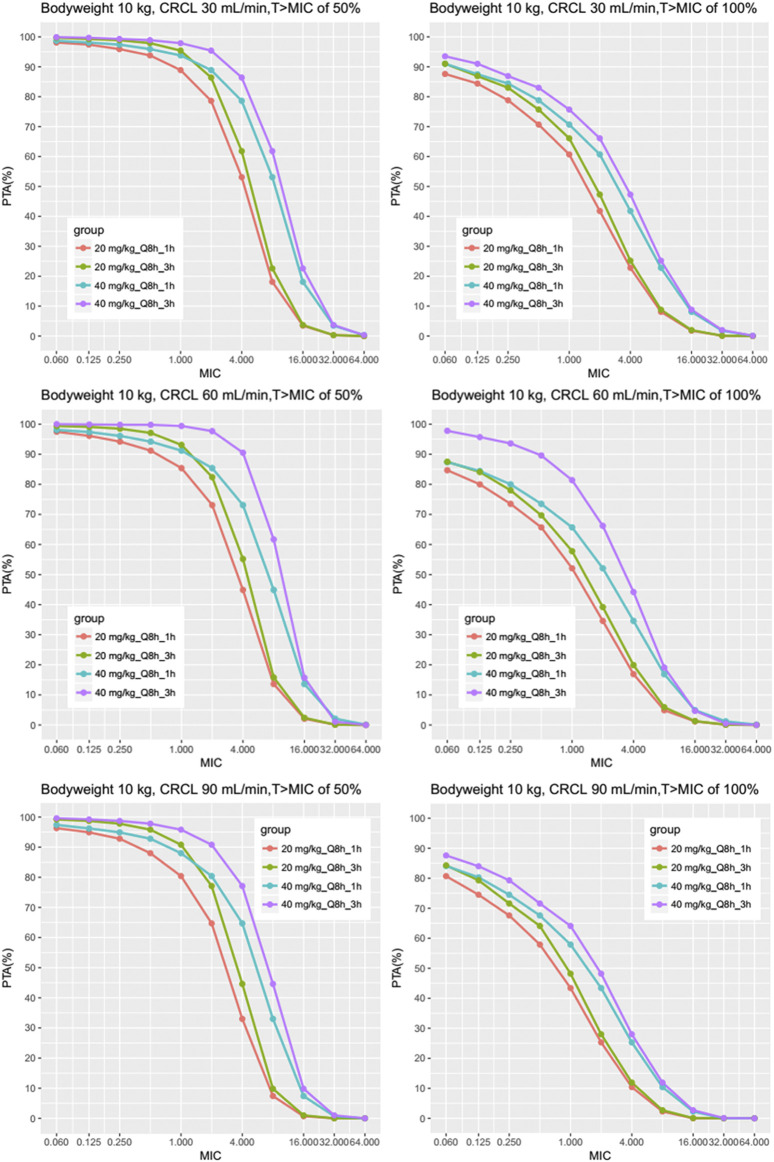
Results of the Monte Carlo simulation with the fractional target attainments against a range of MICs for body weight 10 kg. The meropenem dosing regimens were as follows: 20 mg/kg every 8 h as a 1-h infusion, 40 mg/kg every 8 h as a 1-h infusion, 20 mg/kg every 8 h as a 3-h infusion by two steps as described previously and 40 mg/kg every 8 h as a 3-h infusion by two steps. Results of different eCRCL were shown and the PTA was evaluated for MICs mainly between 0.25 and 16 mg/L.

In light of silico simulation, the PTA against different MIC for different clinical scenarios were presented in Supplementary Datasheet S3. The PTA against MIC for typical patients of 10 kg is plotted in Figure 4. Administration of 20 mg/kg of meropenem for either 1 h or 3 h every 8 h, results in a PTA (%T > MIC of 50%) approximately 95%, for a MIC of 0.125 mg/L, in children with normal renal function (eCRCL 90 ml/min) and renal impairment receiving CRRT. A gradual decline in target attainment was then observed as the MICs increased.

The PTA against MIC for other scenarios (e.g., bodyweight of 5, 20, and 30 kg) are shown in Supplementary Datasheet S3, which suggested similar results (See Supplementary Datasheet S3: PTA of different MICs and body weight for all scenarios). Table 5 and Table 6 show the PTA for different infusion durations of meropenem at the dose of 20 mg/kg or 40 mg/kg every 8 h using MIC of 2 mg/L. Table 7 describe the suggested dosing regimens according to the renal function, body weight and MIC values.

**TABLE 5:** Percentage of children attaining 50% T > MIC according to renal function.

	20 mg/kg q8h 1 h	20 mg/kg q8h 3 h	40 mg/kg q8h 1 h	40 mg/kg q8h 3 h
eCRCL 30 ml/min/1.73 m^2^				
BW 5 kg	69.9	80.9	83.8	92.3
BW 10 kg	78.6	86.4	88.9	95.4
BW 20 kg	85.1	92.3	93.5	97.6
BW 30 kg	87.7	93.8	94.6	98.0
eCRCL 60 ml/min/1.73 m^2^				
BW 5 kg	62.1	74.5	78.8	89.1
BW 10 kg	73.1	82.4	85.4	97.7
BW 20 kg	80.2	87.9	90.0	95.9
BW 30 kg	83.6	91.3	92.2	97.0
eCRCL 90 ml/min/1.73 m^2^				
BW 5 kg	53.8	67.6	73.6	85.2
BW 10 kg	64.7	77.1	80.4	90.8
BW 20 kg	74.3	84.1	87.0	93.9
BW 30 kg	79.0	87.1	89.5	95.9

Notes: Percentages indicate the PTA for different infusion durations of meropenem at the dose of 20 mg/kg or 40 mg/kg every 8 h. MIC of 2 mg/L has been used as reference, corresponding to the EUCAST breakpoint for Enterobacteriaceae and *P. aeruginosa*.

**TABLE 6:** Percentage of children attaining 100% T > MIC according to renal function.

	20 mg/kg q8h 1 h	20 mg/kg q8h 3 h	40 mg/kg q8h 1 h	40 mg/kg q8h 3 h
eCRCL 30 ml/min/1.73 m^2^				
BW 5 kg	31.4	36.1	49.6	55.2
BW 10 kg	41.8	47.3	60.7	66.1
BW 20 kg	54.1	59.2	70.0	73.8
BW 30 kg	60.4	64.5	74.1	77.4
eCRCL 60 ml/min/1.73 m^2^				
BW 5 kg	24.5	27.6	41.3	46.4
BW 10 kg	34.6	39.2	52.1	66.2
BW 20 kg	45.9	50.3	64.2	67.9
BW 30 kg	52.0	56.9	69.0	72.1
eCRCL 90 ml/min/1.73 m^2^				
BW 5 kg	18.4	21.1	31.8	36.8
BW 10 kg	25.3	28.0	43.4	48.2
BW 20 kg	35.8	39.5	54.6	59.5
BW 30 kg	41.4	46.3	61.1	65.7

Notes: Percentages indicate the PTA for different infusion durations of meropenem at the dose of 20 mg/kg or 40 mg/kg every 8 h. MIC of 2 mg/L has been used as reference, corresponding to the EUCAST breakpoint for Enterobacteriaceae and *P. aeruginosa*.

**TABLE 7:** Dosing regimen suggestions.

Dosing 20 mg/kg q8h for 1h infusion (for a target defined as 50% time > MIC, PTA > 70%)is suggested in the following situation:
● MIC < 1 mg/L
● MIC 1–2 mg/L, body weight (BW) 20–30 kg
● MIC 1–2 mg/L, BW 5–10 kg, eCRCL<30 ml/min with CVVHDF
● MIC 1–2 mg/L, BW 10–20 kg, eCRCL 30–60 ml/min with CVVHDF
Dosing 20 mg/kg q8h for 1 h infusion (for a target defined as 100% time > MIC, PTA > 70%)is suggested in the following situation:
● MIC < 1 mg/L, BW 20–30 kg and eCRCL <30 ml/min with CVVHDF
● MIC < 1 mg/L, BW 30 kg and eCRCL 30–60 ml/min with CVVHDF
Dosing 40 mg/kg q8h for two-step 3h infusion when MIC > 4 mg/L
In other cases, if the dosage of meropenem was 20 mg/kg q8h, an extended two-step method was required to attain a PTA > 70%. If meropenem 40 mg/kg q8h was used for infusion, an infusion duration of 1 h could result in a PTA > 70%

## Discussion

To the best of our knowledge, there have been few studies reporting on meropenem PK/PD in septic children receiving ECLS. Our data suggested that bodyweight and eCRCL affect the PK/PD target attainment. CRRT intensity and ECMO did not influence the PK/PD parameters significantly relating to the non-intensive CRRT setting and low sieving coefficient. We recommended optimal regimen of meropenem depending on different MICs, body weight and eCRCL for septic children. Saito et al. (Saito et al., 2021)reported that renal function, the systemic inflammatory response syndrome score for the clearance, and the use of CRRT for the central volume of distribution were identified as significant covariates influencing meropenem PK. Our research was different in three aspects: 1) The enrolled children were more critically ill with PRISM score ranging 31-42.5 and mortality rate 24.0% (6/25); 2)We tested the meropenem concentrations in the effluent fluid and gave details of the CRRT modalities and density because these data was important for the PK/PD discrepancy of meropenem. Theoritically meropenem can easily get through the hemodialysis membrane but the actual concentrations in the effluent fluid were not so high as imagined. That result was related to the specific feature of the AN69 hemofilter which the recommended regimens based on.

Our study showed large inter-individual variability in meropenem concentration which was already reported in other adult cohorts (Ehmann et al., 2017) (See Supplementary Datasheet S2: The original data chart of enrolled children). There haven’t been any definite dosing regimen suggestions of meropenem for septic adults or children administrated with ECLS or not. Ehmann et al. identified larger inter-individual variability on the PK parameter clearance than on Vd (Ehmann et al., 2017), which was in accordance with our results. Theoretically, Vd increases during ECMO and CRRT because of larger extracorporeal circuit volume, but in fact, it has little effect on meropenem plasma concentration. The characteristics of the AN69 hemofilter lead to a decrease in the CL of Meropenem, which ultimately determines the change in the recommended dosing regimen. We have previously reported the PK parameters of a group of children receiving 20 mg/kg q8h infusion ^15^, but the studied population, the dose regimens and methodology here in this study was different, then we established the pharmacokinetic model for the enrolled population. Therapeutic drug monitoring (TDM) can provide evidence for β-lactam antibiotics effects. Meropenem has a maximal efficacy when its concentration is one to four times above the minimum inhibitory concentration (%T > 1-4*MIC) throughout the dosing interval to achieve adequate therapeutic antibiotic concentrations and exposure (Turnidge 1998; Mattoes et al., 2004). Our study found that the main influencing factor of PTA was eCRCL.

The impacts of ECMO and CRRT are different due to the low meropenem adsorption in the ECMO circuit and the high dialysate rate under CRRT. This is mainly due to the chemical properties of meropenem. Hahn et al reviewed pharmacokinetic changes of antibiotics during ECMO in critically ill adult patients reporting that ECMO had no effect on meropenem PK (Rapp et al., 2020). Moreover, Shekar et al performed a matched cohort study on combined effects of ECMO and CRRT on meropenem PK in adults and found that decreased CL on ECMO compensates ECMO and critical-illness related increased volume of distribution (Vd) (Shekar et al., 2014). ECMO and CRRT can increase Vd because of the extracorporeal circuits, but our study found the influence on meropenem concentration was weaker than the hemofilters reported in the past. The characteristics of AN69 hemofilter decreased CL of CRRT, and additionally, the Extracorporeal Life Support Organization (ELSO) registration data showed 44% of V-A ECMO patients had decreased renal function and needed RRT support (Organization ELS, 2013), leading to decreased eCRCL, which resulted in changes of recommended dosing regimen of meropenem in septic children receiving ECLS.

In this study, all the 13 children undergoing ECMO received V-A ECMO, and 4 of them demanded CRRT. Children in our study receiving ECMO had similar CL [11.59 (5.92–20.19) vs 13.51 (3.71-20.80)L/h, *p* = 0.98] compared to controls. No significant changes in PK exposures were observed in children with sepsis who were receiving ECMO. Moreover, the CL of ECMO population was higher than the CL in ECMO adults [11.59 (5.92–20.19) vs 7.9 ± 5.9 L/h] in Shekar’s study due to the higher eCRCL among our ECMO population [162.5 (110.7–204.5) vs 108 (65–183) ml/min]; however, considering the limited size of the study population, future studies with larger samples are needed to further prove these results.

Meropenem dosing in critically ill adults and children with sepsis and CRRT is complicated. Many factors, such as pathophysiological status, renal function, and the intensity of CRRT, can affect the drug’s pharmacokinetics (PK) and pharmacodynamics (PD) of meropenem. The approved standard antibiotic dosing regimens for adults (normal renal function [RF]) include 500 mg or 1,000 mg, which are administered as short-term infusions every 8 h; for other indications, doses up to 2000 mg are recommended (Datapharm 2017). Kritsana et al. found that meropenem administration via 3 h infusion using the same dose improved the PTA in 14 children suffering from severe infection (Kongthavonsakul et al., 2016). Moreover, Rapp et al. recommended a continuous infusion of 60 and 120 mg/kg per day as the adequate dosing regimen to attain the target of 50%fT > MIC and 100%fT > MIC when MIC is > 4 mg/L without risk of accumulation, except for children with severe renal failure (Rapp et al., 2020). Besides, the pharmacokinetics of meropenem in septic children with different renal functions who receive CRRT still remains poorly understood. Previous studies have indicated that RF is the major cause of variability in meropenem exposure (Christensson et al., 1992; Goncalves-Pereira et al., 2014; Jaruratanasirikul et al., 2015; Roberts et al., 2015; Roehr et al., 2015; Kees et al., 2016; Tsai et al., 2016; Rapp et al., 2020), and, consequently it influences the attainment of specific target concentrations (Isla et al., 2008; Roberts et al., 2009; Alobaid et al., 2016). Rapp et al. found that the age and related renal maturation effect were not influential covariate on meropenem CL since eGFR varies because of the critical illness rather than age in this specific population.

Theoretically, CRRT should increase Vd and CL. Yet, in our study, the CRRT group did not show a significant increase of total CL, the reasons of which had been discussed in our published literature (Wang et al., 2021). The patient pathological and physiological conditions can influence the sieving coefficient, molecular weight of the drug, and properties of the hemofiltration membrane (Clark et al., 2018). The specific septic population and physical properties of hemofilter may also affect the drug dose. A more detailed study focusing on the CRRT population could help to identify CRRT dialysate and filtration flow rate effect on dosing requirements.

This study has several key limitations. Firstly, the sample size is small and we need more data on ECMO for septic children; because of the limited data we did not stratify data on the pediatric septic population receiving CRRT. Secondly, we only assessed the effect of 1 h infusion and two-step 3 h infusion. Although continuous infusion is usually used for meropenem when MIC is > 4 mg/L, we did not assess the effect of it because the side effect of 24-h continuous infusion for example renal function damage should be investigated cautiously. Our following study will focus on pediatric sepsis under CRRT to investigate the impact of CRRT density on PK changes among children under acute kidney injury (AKI) and non-AKI conditions.

## Conclusion

The differences in the pharmacokinetics/pharmacodynamics of meropenem in children with sepsis are affected by the bodyweight and endogenous creatinine clearance. The results of this study provide a rationale for dosage adjustment of meropenem and therapeutic drug monitoring in children with sepsis during extracorporeal support.

## Data Availability

The original contributions presented in the study are included in the article/Supplementary Material, further inquiries can be directed to the corresponding authors.

## Abbreviations

CRRT, continuous renal replacement therapy.
