# Supplementary material for: Optimized Dosing Regimens of Meropenem in Septic Children Receiving Extracorporeal Life Support
Source: Front Pharmacol. 2021 Aug 24;12:699191. doi: 10.3389/fphar.2021.699191 (PMC8421735; doi:10.3389/fphar.2021.699191)
Supplement: Supplementary file 3 [file DataSheet1.docx]

**Bioanalytical method for meropenem concentration**

Meropenem was determined in human plasma based on internal standard method by high performance liquid chromatography and electrospray tandem mass spectrometry (HPLC-MS/MS). The analysis column was a Ultimate AQ-C18 column (4.6 mm×250 mm，5 μm). Meropenem and antipyrine (internal standard, IS) were eluted by mobile phase consisted of 0.1% methanoic acid and acetonitrile (80:20, v/v) delivered at a flow rate of 0.9 mL/min. The ESI(+)-MS/MS in Multiple reaction monitoring (MRM) mode was applied to the determination of meropenem (m/z：384.2→141.1) and antipyrine (189.4→104.2).

Stock solution of IS (1 mg/mL) and standard solution of meropenem (1 mg/mL) were prepared in methanol. Stock solution of IS were diluted 10000 times with acetonitrile, getting the standard solution of IS (0.1 μg/mL). The sample pretreatment is shown as follows: A 300 µL aliquot of IS standard solution was added into 100 µL of human plasma. The samples were mixed by vortex and centrifuged at 10000 rpm for 10 min. The supernatant was transferred to a test tube and evaporated to dryness under a stream of nitrogen at room temperature. The residue was reconstituted in 50 µL of mobile phase, then 5 µL was subjected to HPLC-MS/MS analysis. The calibration standards were prepared by spiking standard solution of meropenem into blank human plasma to give a calibration range of 0.3-80 µg/mL (0.3, 0.8, 20, 40, 60, and 80 µg/mL). The IS standard solution was added to each calibration standard in the same way as the sample pretreatment.

The limit of quantification (LOQ) and limit of detection (LOD) were determined by analyzing multiple concentrations (0.1, 0.3, 0.5, and 1.0 µg/mL) of meropenem in human plasma. The LOQ was determined when the signal-to-noise ratio (S/N) is over 10, and the LOD was determined when S/N is over 3.

The method has been shown to be precise and accurate by the five replicate determinations of human plasma containing meropenem at three concentrations (0.8, 20, and 60 µg/mL) in a single batch (intra-assay precision) and three different batches (inter-precision). The overall intra-assay precision for meropenem for the low, middle and high QCs (quality controls) was 16.11%, 4.77% and 2.27% RSD, respectively. The overall inter-assay precision for the low, middle and high QCs was 16.36%, 9.69% and 2.44% RSD, respectively. The accuracy for the low, middle, and high QCs was 19.57%, 4.77% and 2.27% RSD, respectively. And the recovery for the low, middle and high QCs was 81.92%, 100.67% and 105.89%, respectively.

To evaluate extraction recovery, three sets of standards containing meropenem at 0.8 and 60 μg/mL were analyzed. Set A was prepared in human plasma as describe earlier. Set B (neat set) was prepared in methanol. Set C is prepared as follows: A 300 µL aliquot of IS standard solution was added into 100 µL of human plasma. The samples were mixed by vortex and centrifuged at 10000 rpm for 10 min. The supernatant was transferred to a test tube and evaporated to dryness under a stream of nitrogen at room temperature. The residue was reconstituted in 50 µL of mobile phase containing meropenem (1.6 and 120 μg/mL), then 5 µL was subjected to HPLC-MS/MS analysis. Meropenem solution prepared in methanol and human plasma were compared to evaluate matrix effects.
